# Pharmacokinetics of Daprodustat and Metabolites in Individuals with Normal and Impaired Hepatic Function

**DOI:** 10.1002/cpdd.1090

**Published:** 2022-03-30

**Authors:** Kelly M. Mahar, Bonnie C. Shaddinger, Bandi Ramanjineyulu, Susan Andrews, Stephen Caltabiano, Alistair C. Lindsay, Alexander R. Cobitz

**Affiliations:** ^1^ Clinical Pharmacology Modeling & Simulation GlaxoSmithKline Collegeville Pennsylvania USA; ^2^ Medicine Delivery Unit GlaxoSmithKline Collegeville Pennsylvania USA; ^3^ Biostatistics GlaxoSmithKline Bangalore India; ^4^ Clinical Science & Study Operations GlaxoSmithKline Collegeville Pennsylvania USA

**Keywords:** anemia, daprodustat, hepatic impairment, pharmacodynamics, pharmacokinetics

## Abstract

Daprodustat is a hypoxia‐inducible factor‐prolyl hydroxylase inhibitor in development for treatment of anemia of chronic kidney disease. We evaluated the role of hepatic impairment on daprodustat pharmacokinetics, pharmacodynamics, and tolerability. Participants with mild (Child‐Pugh Class A, score 5‒6) and moderate (Child‐Pugh Class B, score 7‒9) hepatic impairment and matched healthy controls were administered single 6‐mg doses of daprodustat. Exposure parameters were determined for daprodustat and its six metabolites. Comparisons resulted in 1.5‐ and 2.0‐fold higher daprodustat C_max_ and area under the curve (AUC) exposures in participants with mild and moderate hepatic impairment, respectively, versus controls; C_max_ in mild hepatic impairment was comparable to controls. Similarly, aligned with parent drug, unbound daprodustat C_max_ and AUC exposures increased 1.6‐ to 2.3‐fold in hepatic‐impaired participants versus controls, and metabolite exposures were 1.2‐ to 2.0‐fold higher in participants with hepatic impairment. Erythropoeitin (EPO) baseline‐corrected AUC exposures were between 0.3‐fold lower and 2.2‐fold higher in matched controls versus hepatic‐impaired participants. No serious or study drug‐related adverse events were reported. Daprodustat exposure was increased in participants with moderate and mild hepatic impairment compared with matched controls; however, no meaningful differences in EPO were observed and no new safety concerns were identified (ClinicalTrials.gov: NCT03223337).

Anemia is a common complication in patients with advanced chronic kidney disease (CKD), involving multiple mechanisms including relative erythropoietin deficiency and impaired iron absorption and utilization.^1^, ^2^ The standard treatment for anemia of CKD includes the use of recombinant human erythropoietin (rhEPO) and its analogs to increase hemoglobin levels.^3^, ^4^ However, treatment with rhEPO or its analogs may be associated with an increased risk of major cardiovascular events (eg, stroke, myocardial infarction).^5^, ^6^, ^7^, ^8^

Daprodustat (GSK1278863) is a small‐molecule, oral hypoxia‐inducible factor‐prolyl hydroxylase enzyme inhibitor (HIF‐PHI) currently in development for the treatment of anemia of CKD.^9^, ^10^, ^11^, ^12^, ^13^ Daprodustat mimics the effects of hypoxia to stimulate erythropoiesis by increasing EPO, and subsequently hemoglobin levels, without exposing the patient to supraphysiologic EPO levels.^11^, ^13^, ^14^, ^15^

Clinical studies have shown that daprodustat achieves or maintains target hemoglobin levels in patients with CKD, including both patients on dialysis and those not on dialysis, irrespective of previous rhEPO treatment.^11^, ^12^, ^13^ Across these studies, the adverse event (AE) profile with daprodustat was consistent with the study population.^11^, ^12^, ^13^

Nonclinical studies have identified that daprodustat is metabolized via cytochrome (CYP) P450 2C8 enzyme and inhibition, and drug interaction studies have provided evidence that daprodustat is also metabolized by CYP2C8 in vivo.^15^, ^16^ The structure of daprodustat and its six predominant metabolites (i.e. metabolites present in the highest concentration in circulation) have previously been reported, with similar in vitro inhibitory potency against PHDs and selectivity against collagen prolyl hydroxylase (CP4H) and factor‐inhibiting HIF (FIH) among the predominant metabolites relative to daprodustat.^15^, ^17^, ^18^ Additionally, preclinical repeat‐dose toxicity studies suggest similar toxicologic profiles compared with daprodustat.^19^ Characterization of the absorption, distribution, metabolism, and excretion showed low urine recovery of the parent drug, indicating that daprodustat and its metabolites are mainly eliminated via hepatobiliary and fecal routes.^20^, ^21^

The primary objective of this study was to compare plasma pharmacokinetic (PK) parameters of daprodustat and its six predominant metabolites in participants with hepatic impairment versus healthy matched controls following oral administration of a single 6‐mg dose of daprodustat. Secondary objectives were to investigate the pharmacodynamic effect (ie, EPO production) and tolerability following a single dose of daprodustat in this population.

## Methods

### Study Design

This was an open‐label, nonrandomized, parallel, adaptive study to examine daprodustat PK and pharmacodynamics (PD) in adults with moderate hepatic impairment (Child‐Pugh Class B, score of 7‒9 points, inclusive) compared to matched healthy controls in Part 1, and adults with either mild or severe hepatic impairment (depending on results from Part 1) compared to matched healthy controls in Part 2 (ClinicalTrials.gov identifier NCT03223337). The study took place at two centers (Orlando Clinical Research Center, Orlando, Florida, and University of Miami, Division of Clinical Pharmacology, Miami, Florida) from July 2017 to August 2018 and was conducted according to the recommendations of Good Clinical Practice and the Declaration of Helsinki (2013). The research protocol was approved by the relevant national, regional, or investigational center ethics committee or institutional review boards: IntegReview IRB, Austin, Texas, and the Western Institutional Review Board, Puyallup, Washington. Written informed consent was obtained from all participants prior to initiation of the study.

All study participants received a single, oral, 6‐mg dose of daprodustat whilst in the fasted state, followed by sampling for PK and PD assessments (see Blood Sample Collection and Analysis). Plasma samples were analyzed for concentrations of daprodustat, its six predominate metabolites (GSK2391220 [M2], GSK2506104 [M3], GSK2487818 [M4], GSK2506102 [M5], GSK2531398 [M6], and GSK2531401 [M13]), and EPO. A follow‐up visit occurred 10‒14 days after administration of the study drug.

The study was divided into two parts. In Part 1, participants with moderate hepatic impairment (Cohort 1) were matched in gender, age (±10 years), and body mass index (BMI) (±15%) with healthy control participants (Cohort 2). Following completion of Part 1, an interim analysis of daprodustat exposure was performed to determine whether participants with mild or severe hepatic impairment would be enrolled in Part 2 of the study. The total plasma area under the concentration‐time curve (AUC) of daprodustat extrapolated to infinite time (AUC_0‐inf_) was increased (geometric least‐squares ratio [GMR]) by 2.0‐fold in the moderate hepatic impairment cohort in Part 1, therefore, in accordance with predefined decision criteria, Part 2 enrolled participants with mild hepatic impairment (Child‐Pugh Class A, score of 5‒6 points, inclusive) in Cohort 3. Gender‐, age (±10 years)‐, and BMI (±15%)‐matched healthy control participants were enrolled in Cohort 4.

### Eligibility Criteria

This study included adults at least 18 years of age. Eligibility criteria included hemoglobin values of ≤16.0 g/dL for males and ≤14.0 g/dL for females, body weight ≥45 kg, and BMI 18.0–40.0 kg/m^2^ (inclusive). Participants with hepatic impairment (of any etiology) had to be clinically stable for at least 1 month prior to and throughout screening. Participants with mild hepatic impairment had a Child–Pugh (Class A) score of 5–6 and previous confirmation of liver disease, while participants with moderate hepatic impairment had a Child–Pugh (Class B) score of 7–9 and previous confirmation of liver cirrhosis. The inclusion criteria did not mandate the need for participants to have renal disease or anemia. Key exclusion criteria included a history of myocardial infarction, acute coronary syndrome, stroke, or transient ischemic attack within the 12 weeks before study enrollment and chronic inflammatory joint disease (see Supporting Information for complete exclusion criteria).

### Study Population

The study enrolled 37 participants, all of whom completed the study. Part 1 of the study included 16 participants, eight with moderate hepatic impairment (Cohort 1) matched to 8 healthy controls (Cohort 2). The protocol was an adaptive study design, dependent on the results observed in Part 1 in the moderate hepatic impairment cohort. If the geometric mean total plasma AUC_0‐inf_ of daprodustat was increased in Part 1 by ≥2‐fold in moderately impaired participants relative to matched controls, Part 2 would evaluate daprodustat pharmacokinetics in participants with mild impairment (Child‐Pugh score of 5–6, n = 8) and in matched control participants (n = 8). Alternatively, if the increase in daprodustat exposure was <2‐fold in moderately impaired participants relative to matched controls, Part 2 would evaluate daprodustat pharmacokinetics in participants with severe impairment (Child‐Pugh score of 10–13, n = 8) and in matched control participants (n = 8). Part 2 of the study included 21 participants, 12 with mild hepatic impairment (Cohort 3) matched to nine healthy controls (Cohort 4).

The safety population included all participants who received at least one dose of study medication. The PD population included all participants in the safety population who had at least 1 PD assessment. The PK population included all participants in the safety population for whom a PK sample was obtained and analyzed. PK and PD samples that may have been affected by protocol deviations were reviewed by the study team to determine if the sample was to be excluded from the analyses.

All 37 participants were included in the safety population and PD population. Five participants’ PK samples thawed during shipment and were not analyzable and were therefore excluded from the PK population. The final PK population included 32 participants (Cohort 1, n = 8; Cohort 2, n = 8; Cohort 3, n = 8; Cohort 4, n = 8). In Part 1, there was evidence of biological matrix interference that could not be resolved for 2 of the metabolites, resulting in no reported PK data for metabolite GSK2506104 [M3] and qualified data reported for metabolite GSK2487818 [M4].

### Study Endpoints

The following PK parameters were measured: AUC_0‐inf_, AUC from time zero (predose) to last time of quantifiable concentration within a subject across all treatments (AUC_0‐t_), maximum observed concentration (C_max_), terminal phase half‐life (t_1/2_), and time of occurrence of C_max_ (t_max_). In addition, unbound concentration and unbound fraction of daprodustat and its predominant metabolites in plasma were evaluated.

Pharmacodynamic endpoints were the maximum observed EPO concentration (C_max_, _EPO_), time of occurrence of C_max_, _EPO_ (t_max_, _EPO_), and EPO AUC from time zero (predose) to the last time of quantifiable EPO concentration within a participant across all treatments (AUC_0‐t, EPO_). The safety and tolerability of daprodustat, including AEs, clinical laboratory tests (hematology, clinical chemistry, and urinalysis), vital signs, electrocardiograms (ECGs), and physical examination findings, were also assessed.

### Blood Sample Collection and Analysis

Blood samples for PK and PD analyses were collected at predose and 0.5, 1, 1.5, 2, 3, 4, 6, 8, 10, 12, 24, 36, and 48 hours postdose. For protein binding assays, samples were collected at predose and 3, 12, and 24 hours postdose.

Whole‐blood samples for PK and protein binding analysis were collected into K_3_EDTA tubes, gently inverted several times, and immediately placed on water ice. Samples were then centrifuged at 1500 **×** g for 15 minutes; the supernatant plasma was transferred to Nunc tubes and stored at −20°C before shipment. Samples for daprodustat and specific assay metabolite measurements were shipped frozen to PPD Laboratories (PPD) (Middleton, Wisconsin). Samples for EPO measurements were shipped frozen to Q2 Solutions (Valencia, California).

### Total Plasma PK Concentration Analysis

Plasma samples were analyzed for daprodustat and its six metabolites by PPD using two validated analytical methods based on solid phase extraction (SPE), followed by high‐performance liquid chromatography with tandem mass spectrometric detection (HPLC/MS/MS) analysis using an electrospray interface operated in negative ion mode, as described previously.^14^, ^17^ For Part 1 of the study, a 250‐μL aliquot of EDTA plasma was extracted using SPE (Biotage Evolute ABN). The lower limit of quantification (LLQ) and higher limit of quantification (HLQ) were 5.00 and 2500 pg/mL, respectively, for daprodustat, and 10.0 and 5000 pg/mL, respectively, for all metabolites. The data accuracy (% bias range −4.15% to 2.56% for daprodustat and −7.79% to 19.6% for all metabolites) and precision (%CV of 2.04%–11.5% for daprodustat and 0.797%–11.2% for all metabolites) met acceptable limits as recommended by the FDA in their bioanalytical method validation guidelines.^22^

For Part 2 of the study, a 250‐μL aliquot of EDTA plasma was extracted using SPE (Waters Oasis MAX), the LLQ and HLQ were 5.00 and 2500 pg/mL, respectively, for daprodustat, and 10.0 and 5000 pg/mL, respectively, for all metabolites. The data accuracy (% bias range −5.86% to 2.71% for daprodustat and −0.331% to 5.54% for all metabolites) and precision (%CV of 1.29%–4.48% for daprodustat and 1.40%–8.35%) met acceptable limits as recommended by the FDA in their bioanalytical method validation guidelines.^22^

### Unbound Plasma PK Concentration Analysis

Unbound concentrations in plasma of daprodustat and its six metabolites from the 3, 12, and 24 hours postdose were determined by PPD via a rapid equilibrium dialysis procedure. Plasma samples, collected from whole blood in K_3_EDTA tubes, were dialyzed to 1:1 plasma:phosphate‐buffered saline (PBS) and analyzed for concentrations of daprodustat and its six predominate metabolites (GSK2391220 [M2], GSK2506104 [M3], GSK2487818 [M4], GSK2506102 [M5], GSK2531398 [M6], and GSK2531401 [M13]) using a validated method. The degree of nonspecific binding for daprodustat and each metabolite was evaluated, and recovery was determined at >95% for each analyte. The optimal time of dialysis was determined to be 24 hours to ensure equilibrium was achieved. Three concentrations of each analyte (15.0, 25.0, 50.0 ng/mL for daprodustat and 22.5, 37.5, and 75.0 ng/mL for each metabolite) were evaluated to determine the effect of concentration on binding, and plasma protein binding was not found to be concentration‐dependent across the aforementioned concentrations.

A 300‐μL plasma matrix aliquot was added to the donor side of the dialysis unit and 500‐μL blank PBS aliquot added to the receptor side of the dialysis unit. The unit was covered with sealing tape and incubated on an orbital shaker in an air incubator set to 37°C for 24 hours. At the end of dialysis, 250 μL of the dialyzed plasma donor was diluted with 250 μL of undialyzed blank PBS. Separately, 250 μL of the dialyzed PBS receptor was diluted with 250 μL of undialyzed blank plasma. A 250‐μL matrix aliquot was fortified with 20 μL of 25.0/25.0/50.0/50.0 ng/mL [13C5 15N]‐daprodustat/[13C5 15N]‐GSK2391220/[13C5 15N]‐GSK2531398/[13C5 15N]‐GSK2531401 internal standard working solution. Analytes were isolated through SPE (Water Oasis MAX 30 mg SPE plates) and reconstituted with 200 μL of water/acetonitrile/formic acid [700/300/1] and analyzed via HPLC with MS/MS detection using negative ion electrospray. The assay was validated over the daprodustat concentration range of 0.0100–10.0 ng/mL and GSK2391220 (M2), GSK2506104 (M3), GSK2487818 (M4), GSK2506102 (M5), GSK2531398 (M6), and GSK2531401 (M13) concentration range of 0.0150–15.0 ng/mL in 1:1 human plasma:PBS. Quality controls at the low‐, mid‐, and high‐levels were analyzed in duplicate in each evaluation run to determine run acceptance.

### EPO PD Concentration Analysis

Whole‐blood samples were collected into K_2_EDTA tubes, gently inverted several times, and immediately placed on water ice. Samples were then centrifuged at 1600 **×** g for 10 minutes; the supernatant plasma was transferred to conical bottom polypropylene centrifuge tubes using a transfer pipette. These samples were then centrifuged for 10 minutes for complete platelet removal. All supernatant plasma was transferred with a pipette (without disturbing the pellet at the bottom) into labeled Nunc tubes and stored at −20°C or below before shipment. Samples for EPO measurements were shipped frozen to Q2 Solutions. The Quantikine IVD Human EPO Immunoassay (R&D Systems Inc., Catalog No. DEP00) analytical method was used, which is based on a double‐antibody, sandwich enzyme‐linked immunosorbent assay (ELISA) in 96‐well format. A standard curve was constructed to show the direct correlation between EPO concentration (mIU/mL) and instrument response (optical density [OD]). The microplate wells, precoated with monoclonal (mouse) EPO‐specific antibody, were incubated with each specimen or standard; EPO binds to the immobilized antibody on the plate. After removing excess specimen or standard, wells were incubated with an anti‐EPO polyclonal (rabbit) antibody conjugated to horseradish peroxidase. During the second incubation, the antibody enzyme conjugate binds to the immobilized EPO. Excess conjugate was removed by washing, a chromogen was added into the wells, and was oxidized by the enzyme reaction to form a blue‐colored complex. The reaction was stopped by the addition of acid, which turns the blue to yellow. The amount of color generated is directly proportional to the amount of conjugate bound to the EPO antibody complex and is directly proportional to the amount of EPO in the specimen or standard. The absorbance of this complex was measured and a standard curve generated by plotting absorbance versus the concentration of the EPO standards. The EPO concentrations of the unknown specimens were determined by comparing the OD of the specimen to the standard curve spectrophotometrically using a Tecan GENios Pro microplate reader at a primary wavelength of 450 nm and a reference wavelength of 610 nm to correct for optical imperfections in the polystyrene microplate. Absorbance is expressed in terms of OD values at a measurement wavelength of 450 nm with the correction wavelength set at 610 nm (OD450–610). The concentration range of detection was 2.5–200 IU/L, with accuracy and precision estimates falling within 10% for all seven nonzero standards. Samples that produced a signal above the ULOQ were further diluted up to 1:16 in specimen diluent and reassayed.

### Safety Assessments

Laboratory tests were performed at screening, 48 hours postdose, and the follow‐up visit. ECG, physical examination, and vital signs were assessed at screening, day 1, 48 hours postdose, and the follow‐up visit. AEs and SAEs were collected from the start of study treatment until the follow‐up visit.

### Pharmacokinetic and Pharmacodynamic Analysis

Plasma PK concentration‐time data for daprodustat and its six predominant metabolites were analyzed by noncompartmental methods using Phoenix WinNonlin software (Pharsight Corporation, St Louis, Missouri) Version 6.4.

Calculations were based on the actual sampling times recorded during the study. From the plasma concentration‐time data, the following PK parameters were determined for daprodustat and its metabolites as the data permitted: maximum observed plasma concentration (C_max_), time to C_max_ (t_max_), area under the plasma concentration‐time curve (AUC_0‐t_ and AUC_0‐inf_), and apparent terminal phase half‐life (t_½_).

The unbound fraction (fu) was calculated using the total and unbound plasma concentration of daprodustat and its metabolites generated at 3, 12, and 24 hours postdose for both normal and hepatic‐impaired participants using the following formula:

(1) $$fu=C_{unbound}/C_{total}$$

The unbound daprodustat PK parameters (free C_max_ and free AUC_0‐inf_) were derived by multiplying the PK parameter values for total daprodustat by the fu value obtained for each subject. Calculated fu values greater than 1 due to quantified unbound PK concentrations being higher than the total PK concentrations (without evidence of any analytical error) were excluded from calculations for the summary tables given the biologically implausible nature of these data.

Pharmacodynamic parameters were determined from plasma concentration‐time data from EPO measurements for absolute EPO and baseline corrected EPO parameters were calculated from the individual predose EPO measurements. Maximum observed erythropoietin concentration (C_max_, _EPO_), time of the maximum observed erythropoietin concentration (t_max_, _EPO_), and erythropoietin area under the concentration‐time curve from time zero (predose) to the last time of quantifiable concentration (AUC_0‐t, EPO_) were determined using WinNonlin software (Pharsight Corporation, St Louis, Missouri) Version 6.4.

### Statistical Analyses

No formal hypotheses were tested. An estimation approach was used to evaluate the effect of hepatic impairment on the PK of daprodustat. Point estimates of the geometric least squares mean ratio for the PK parameters and associated 90% confidence intervals (CIs) were provided for matched cohort comparisons (hepatically impaired:matched healthy participants). The PK parameters were log‐transformed prior to analysis, and matched cohort comparisons were expressed as ratios on the original scale.

The log‐transformed AUC_0‐inf_, C_max_, and t_1/2_ values for daprodustat and its six predominant metabolites were analyzed separately using analysis of variance (ANOVA), fitting a fixed‐effect term for the cohort. Point estimates and 90% CIs for the cohort comparisons (hepatically impaired vs matched healthy controls) were constructed using the residual variance. PK statistical analyses were performed when sufficient data were available. t_max_ was analyzed nonparametrically using the Mann–Whitney U test (Wilcoxon rank sum test) when possible. The point estimates and 90% CIs for the median differences were derived for hepatic impairment and healthy matched controls based on Hodges–Lehmann estimation. The point estimates and 90% CIs for the median differences were calculated for the cohort difference (hepatically impaired – matched healthy controls). A similar estimation analysis was performed on the absolute EPO and baseline corrected PD parameters.

A sensitivity analysis was performed, in which the log‐transformed AUC_0‐inf_, C_max_, and t_1/2_ values for daprodustat and its six predominant metabolites were analyzed separately using ANCOVA fitting fixed‐effect terms for cohort, gender, age, and BMI. Point estimates and 90% CIs for the differences of interest (hepatically impaired vs healthy matched controls) were constructed using the residual variance.

Safety assessments were summarized descriptively.

### Safety and Tolerability Measures

Laboratory tests were performed at screening, 48 hours postdose, and the follow‐up visit. ECGs, physical examinations, and vital signs were assessed at screening, day 1, 48 hours postdose, and the follow‐up visit. AEs and SAEs were collected from the start of study treatment until the follow‐up visit. Safety and tolerability assessments were summarized descriptively.

## Results

Demographics and baseline characteristics were balanced across all four cohorts (Table 1). Overall, the mean age was 58.6 and 60.5 years in Part 1 and Part 2, respectively. Over 80% of participants were male in both Part 1 and Part 2. The mean Child‐Pugh score was 8 (range 7‒9) in Cohort 1 (moderate hepatic impairment) and 5.8 (range 5‒6) in Cohort 3 (mild hepatic impairment).

**Table 1:** Demographics and Baseline Characteristics

	Part 1			Part 2		
Demographics/baseline Characteristics	Moderate HI (n = 8)	Matched Controls (n = 8)	Total (n = 16)	Mild HI (n = 12)	Matched Controls (n = 9)	Total (n = 21)
Age (years), mean (SD)	59.5 (6.0)	57.6 (7.2)	58.6 (6.5)	61.9 (7.6)	58.7 (8.0)	60.5 (7.7)
Age ranges (years), n (%)						
Adult (18‒64)	6 (75)	7 (88)	13 (81)	9 (75)	7 (78)	16 (76)
≥65‒84	2 (25)	1 (13)	3 (19)	3 (25)	2 (22)	5 (24)
≥85	0	0	0	0	0	0
Sex, n (%)						
Female	1 (13)	1 (13)	2 (13)	2 (17)	2 (22)	4 (19)
Male	7 (88)	7 (88)	14 (88)	10 (83)	7 (78)	17 (81)
BMI (kg/m^2^), mean (SD)	32.4 (3.8)	29.9 (3.2)	31.1 (3.6)	29.8 (4.0)	28.1 (2.6)	29.1 (3.5)
Height (cm), mean (SD)	169 (6.4)	171 (6.0)	170 (6.1)	170 (6.4)	170 (9.7)	170 (7.7)
Weight (kg), mean (SD)	92.6 (10.3)	87.6 (10.9)	90.1 (10.5)	86.8 (15.1)	80.4 (9.0)	84.0 (13.0)
Ethnicity, n (%)						
Hispanic or Latino	4 (50)	5 (63)	9 (56)	9 (75)	7 (78)	16 (76)
Not Hispanic or Latino	4 (50)	3 (38)	7 (44)	3 (25)	2 (22)	5 (24)
Race, n (%)						
Asian, East Asian heritage	0	0	0	1 (8)	0	1 (5)
Black or African American	0	2 (25)	2 (13)	1 (8)	2 (22)	3 (14)
White, Arabic/North African heritage	0	0	0	4 (33)	1 (11)	5 (24)
White, White/Caucasian/European heritage	8 (100)	6 (75)	14 (88)	6 (50)	6 (67)	12 (57)
Child‐Pugh score						
Mean (min, max)	8.0 (7, 9)	NA	NA	5.8 (5, 6)	NA	NA
Baseline erythropoietin (IU/L), mean (SD)	23.3 (32.4)	10.7 (9.0)		20.0 (18.2)	12.7 (10.4)	
Hemoglobin (g/dL), mean (SD)	13.6 (22.3)	14.3 (9.1)		13.9 (8.1)	13.9 (11.1)	
Albumin (g/L), mean (SD)	40.5 (5.5)	45.9 (2.0)		43.1 (3.9)	42.9 (2.1)	
Creatinine (mg/100 mL), mean (SD)	0.71 (0.12)	0.73 (0.12)		0.93 (0.19)	0.90 (0.17)	

BMI, body mass index; HI, hepatic impairment; NA, not applicable; SD, standard deviation.

### Pharmacokinetics

**Figure 1:**
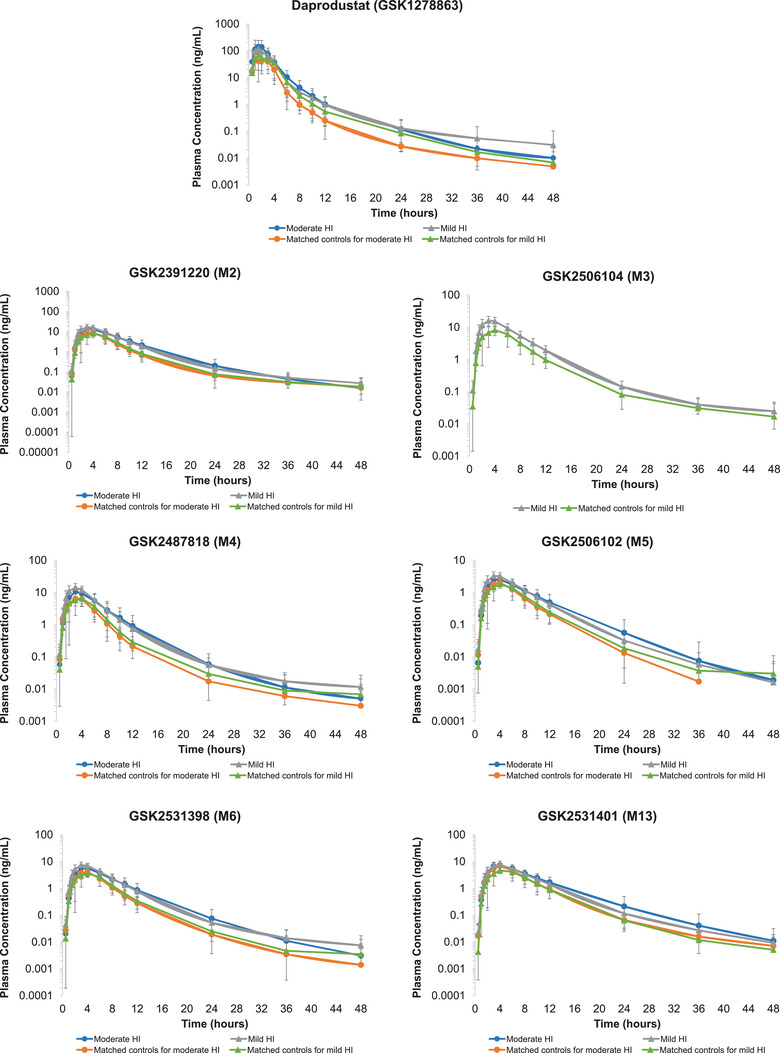
Daprodustat and metabolite plasma concentrations over time. Plasma daprodustat and metabolite concentrations following administration of a single 6‐mg dose of daprodustat on a semilogarithmic scale (mean ± standard deviation). The lower limit of quantification corresponds to 0.005 ng/mL for daprodustat and 0.01 ng/mL for all metabolites. HI, hepatic impairment.

In participants with either mild or moderate hepatic impairment, the plasma PK profiles for daprodustat and its metabolites (Figure 1), following a single oral administration of 6 mg, were higher across the entire time course as compared to matched healthy controls. For daprodustat, the peak exposure to daprodustat (C_max_) was attained (t_max_) at median times of 1.5 hours postdose for participants with moderate hepatic impairment, 2.0 hours for moderate hepatic impairment healthy matched controls, 1.5 hours for participants with mild hepatic impairment, and 1.5 hours for mild hepatic impairment healthy matched controls. The t_1/2_ values for daprodustat were similar across all four groups and the mean ranged between 4.2 and 4.7 hours (Table 2). Statistical analysis of daprodustat PK parameters resulted in a GMR increase of 2.0‐fold in C_max_ (90% CI 1.1‒3.7) and AUC_0‐inf_ (90% CI 1.1‒3.6) in participants with moderate hepatic impairment compared to matched healthy controls in Part 1 of the study. In Part 2 of the study, peak exposure (C_max_) of daprodustat in participants with mild hepatic impairment was comparable to matched healthy controls (GMR 1.0, 90% CI 0.7‒1.4), but the overall exposure (AUC_0‐inf_) had a GMR increase of 1.5‐fold (90% CI 1.0‒2.1) in the mild hepatic impairment participants (Table 2). The variability of systemic exposure to daprodustat (C_max_, AUC_0‐t_, and AUC_0‐inf_), as assessed by the between subject coefficient of variation of the geometric mean (%CVb), was higher for the moderate hepatic impairment group (109%‒111%) than healthy matched controls (31.7%‒51.7%), while the extent of systemic exposure variability to daprodustat was comparable between participants with mild hepatic impairment (ranged between 39.9% and 42.6%) and healthy matched controls (ranged between 35.7% and 37.2%) (Table 2).

**Table 2:** Statistical Analysis of Daprodustat and Metabolite Exposure Parameters after Single Doses of Daprodustat Administered to Participants with Moderate and Mild Hepatic Impairment and Matched Healthy Controls

	Part 1		Part 2	
Analyte Parameter	Moderate HI (n = 8)	Matched Controls (n = 8)	Mild HI (n = 8)	Matched Controls (n = 7^a^)
**Daprodustat**				
AUC_0‐inf_ (h•ng/mL)				
Arithmetic mean (SD)	401 (284)	154 (40.9)	321 (142)	217 (72.3)
Geometric mean (%CVb)	296 (111)	148 (31.7)	300 (39.9)	206 (37.2)
Ratio of geometric LS mean (90% CI)	2.0 (1.1, 3.6)		1.5 (1.0, 2.1)	
C_max_ (ng/mL)				
Arithmetic mean (SD)	187 (129.9)	78 (40.2)	122 (55.3)	118 (40.2)
Geometric mean (%CVb)	140 (108.8)	71 (51.7)	113 (42.6)	112 (35.7)
Ratio of geometric LS mean (90% CI)	2.0 (1.1, 3.7)		1.0 (0.7, 1.4)	
t_max_ (h)				
Median (range)	1.5 (1.0–3.0)	2.0 (1.0–3.0)	1.5 (1.0–3.0)	1.5 (1.0–4.0)
t_1/2_ (h)				
Arithmetic mean (SD)	4.2 (1.3)	4.6 (1.3)	4.7 (1.4)	4.6 (1.9)
Geometric mean (%CVb)	4.0 (33.2)	4.4 (29.8)	4.5 (27.4)	4.3 (38.7)
Ratio of geometric LS mean (90% CI)	0.9 (0.7, 1.2)		1.1 (0.8, 1.4)	
**GSK2391220 (M2)**				
AUC_0‐inf_ (h•ng/mL)				
Arithmetic mean (SD)	87.2 (42.9)	49.1 (15.5)	98.4 (37.9)	48.6 (13.3)
Geometric mean (%CVb)	77.6 (56.9)	47.1 (30.9)	91.4 (44.7)	47.2 (26.9)
Ratio of geometric LS mean (90% CI)	1.6 (1.1, 2.4)		1.9 (1.4, 2.7)	
C_max_ (ng/mL)				
Arithmetic mean (SD)	14.0 (4.9)	10.6 (3.0)	16.8 (5.8)	9.4 (3.3)
Geometric mean (%CVb)	13.0 (46.2)	10.2 (28.7)	15.8 (40.8)	8.9 (40.0)
Ratio of geometric LS mean (90% CI)	1.3 (0.9, 1.8)		1.8 (1.2, 2.5)	
t_max_ (h)				
Median (range)	3.0 (3.0–6.0)	3.5 (2.0–4.0)	3.0 (3.0–4.0)	3.0 (2.0–4.0)
t_1/2_ (h)				
Arithmetic mean (SD)	4.3 (1.1)	6.0 (2.2)	4.9 (1.0)	5.3 (3.0)
Geometric mean (%CVb)	4.2 (26.9)	5.6 (37.1)	4.8 (21.2)	4.7 (49.6)
Ratio of geometric LS mean (90% CI)	0.7 (0.6, 1.0)		1.0 (0.7, 1.4)	
**GSK2506104 (M3)** **^b^**				
AUC_0‐inf_ (h•ng/mL)				
Arithmetic mean (SD)	ND	ND	97.8 (34.6)	51.8 (14.8)
Geometric mean (%CVb)	ND	ND	91.8 (41.0)	50.1 (28.3)
Ratio of geometric LS mean (90% CI)	ND	ND	1.8 (1.3, 2.5)	
C_max_ (ng/mL)				
Arithmetic mean (SD)	ND	ND	16.4 (5.4)	9.4 (3.3)
Geometric mean (%CVb)	ND	ND	15.5 (38.8)	8.9 (37.9)
Ratio of geometric LS mean (90% CI)	ND	ND	1.7 (1.2, 2.4)	
t_max_ (h)				
Median (range)	ND	ND	3.5 (3.0–4.0)	3.0 (2.0–6.0)
t_1/2_ (h)				
Arithmetic mean (SD)	ND	ND	4.7 (0.9)	4.9 (2.7)
Geometric mean (%CVb)	ND	ND	4.7 (20.2)	4.5 (45.0)
Ratio of geometric LS mean (90% CI)	ND	ND	1.0 (0.8, 1.4)	
**GSK2487818 (M4)**				
AUC_0‐inf_ (h•ng/mL)				
Arithmetic mean (SD)	55.6 (32.3)	30.2 (9.6)	67.9 (26.9)	33.0 (10.9)
Geometric mean (%CVb)	47.6 (67.4)	29.0 (30.3)	63.6 (40.1)	31.8 (28.4)
Ratio of geometric LS mean (90% CI)	1.6 (1.1, 2.5)		2.0 (1.5, 2.7)	
C_max_ (ng/mL)				
Arithmetic mean (SD)	11.1 (4.6)	8.3 (2.6)	14.3 (4.5)	8.1 (2.6)
Geometric mean (%CVb)	10.0 (55.0)	8.03 (28.5)	13.6 (34.7)	7.7 (35.6)
Ratio of geometric LS mean (90% CI)	1.2 (0.9, 1.8)		1.8 (1.3, 2.4)	
t_max_ (h)				
Median (range)	3.0 (2.0–6.0)	3.0 (1.5–4.0)	3.0 (2.0–4.0)	3.0 (2.0–4.0)
t_1/2_ (h)				
Arithmetic mean (SD)	2.8 (1.1)	4.5 (3.1)	4.5 (1.9)	4.0 (3.7)
Geometric mean (%CVb)	2.7 (38.3)	3.6 (83.8)	4.1(47.8)	3.1 (88.9)
Ratio of geometric LS mean (90% CI)	0.7 (0.4, 1.2)		1.3 (0.8, 2.4)	
**GSK2506102 (M5)**				
AUC_0‐inf_ (h•ng/mL)				
Arithmetic mean (SD)	18.2 (7.7)	11.7 (3.2)	20.4 (6.5)	11.7 (3.2)
Geometric mean (%CVb)	16.8 (43.9)	11.3 (29.4)	19.4 (36.6)	11.3 (26.7)
Ratio of geometric LS mean (90% CI)	1.5 (1.1, 2.0)		1.7 (1.3, 2.3)	
C_max_ (ng/mL)				
Arithmetic mean (SD)	2.8 (0.8)	2.3 (0.5)	3.4 (1.0)	2.1 (0.7)
Geometric mean (%CVb)	2.7 (34.6)	2.2 (23.0)	3.2 (35.7)	2.0 (33.4)
Ratio of geometric LS mean (90% CI)	1.2 (0.9, 1.5)		1.6 (1.2, 2.2)	
t_max_ (h)				
Median (range)	3.5 (3.0–6.0)	4.0 (2.0–4.0)	3.5 (3.0–4.0)	3.0 (2.0–6.0)
t_1/2_ (h)				
Arithmetic mean (SD)	3.3 (0.9)	2.9 (1.1)	3.6 (1.3)	3.9 (3.0)
Geometric mean (%CVb)	3.2 (23.4)	2.7 (36.4)	3.4 (30.8)	3.3 (56.7)
Ratio of geometric LS mean (90% CI)	1.2 (0.9, 1.5)		1.0 (0.7, 1.5)	
**GSK2531398 (M6)**				
AUC_0‐inf_ (h•ng/mL)				
Arithmetic mean (SD)	37.1 (16.0)	21.6 (6.2)	43.1 (12.1)	21.5 (5.0)
Geometric mean (%CVb)	33.9 (49.2)	20.9 (27.7)	41.5 (31.2)	21.0 (23.5)
Ratio of geometric LS mean (90% CI)	1.6 (1.2, 2.3)		2.0 (1.5, 2.5)	
C_max_ (ng/mL)				
Arithmetic mean (SD)	6.3 (2.0)	4.8 (1.2)	7.6 (1.9)	4.2 (1.2)
Geometric mean (%CVb)	5.9 (41.7)	4.7 (24.1)	7.3 (28.2)	4.0 (31.7)
Ratio of geometric LS mean (90% CI)	1.3 (1.0, 1.7)		1.8 (1.4, 2.4)	
t_max_ (h)				
Median (range)	3.5 (3.0–6.0)	3.5 (2.0–4.0)	3.0 (3.0–4.0)	3.0 (2.0–6.0)
t_1/2_ (h)				
Arithmetic mean (SD)	3.2 (0.9)	3.8 (2.8)	4.0 (1.5)	3.5 (2.6)
Geometric mean (%CVb)	3.1 (26.0)	3.3 (56.3)	3.8 (38.4)	3.0 (57.1)
Ratio of geometric LS mean (90% CI)	1.0 (0.7, 1.4)		1.3 (0.8, 1.9)	
**GSK2531401 (M13)**				
AUC_0‐inf_ (h•ng/mL)				
Arithmetic mean (SD)	54.7 (19.2)	41.3 (12.5)	52.6 (21.2)	33.9 (10.9)
Geometric mean (%CVb)	52.1 (34.0)	39.6 (32.1)	46.7 (66.5)	32.0 (40.9)
Ratio of geometric LS mean (90% CI)	1.3 (1.0, 1.7)		1.5 (0.9, 2.3)	
C_max_ (ng/mL)				
Arithmetic mean (SD)	7.8 (2.8)	7.3 (1.8)	7.6 (2.8)	5.4 (1.9)
Geometric mean (%CVb)	7.4 (35.0)	7.1 (28.0)	6.8 (65.5)	5.1 (43.4)
Ratio of geometric LS mean (90% CI)	1.0 (0.8, 1.4)		1.3 (0.8, 2.2)	
t_max_ (h)				
Median (range)	4.0 (3.0–6.0)	4.0 (3.0–6.0)	4.0 (3.0–4.0)	4.0 (3.0–6.0)
t_1/2_ (h)				
Arithmetic mean (SD)	4.0 (1.0)	4.1 (1.4)	4.4 (0.9)	3.8 (1.5)
Geometric mean (%CVb)	3.9 (22.8)	3.9 (33.1)	4.3 (21.2)	3.6 (34.2)
Ratio of geometric LS mean (90% CI)	1.0 (0.8, 1.3)		1.2 (0.9, 1.6)	

%CVb, between subject coefficient of variation; 90% CI, 90th percentile confidence interval; AUC, area under the curve; AUC_0‐inf_, area under the curve zero to infinity; C_max_, maximum observed concentration; EPO, erythropoietin; HI, hepatic impairment; LS, least squares; ND, not determined; SD, standard deviation; t_1/2_, terminal phase half‐life; t_max_, time of occurrence of C_max_.

Analysis of variance with cohort as a fixed effect was performed on the natural log‐transformed parameters total/free AUC_0‐inf_, total/free C_max_, and t_1/2_.

^a^ One participant from the matched controls for mild HI group was excluded from the table summaries due to missing PK endpoints.

^b^ In Part 1, there was evidence of biological matrix interference that could not be resolved for two of the metabolites, resulting in no reported PK data for metabolite GSK2506104 (M3).

For the predominant daprodustat metabolites GSK2391220 (M2), GSK2487818 (M4), GSK2506102 (M5), GSK2531398 (M6), and GSK2531401 (M13) and GSK2506104 (M3, Part 2 only), following a single oral administration of 6 mg of daprodustat, t_max_ was attained between 3.0 and 4.0 hours postdose (median) for all cohorts. The t_1/2_ was comparable for each of the metabolites across all groups and all were found to have mean t_1/2_ values ≤6.0 hours following a single dose of daprodustat (Table 2). Metabolite comparisons for C_max_ and AUC_0‐inf_ revealed a GMR increase between 1.2‐ and 1.6‐fold in participants with moderate hepatic impairment as compared to matched healthy controls, except for C_max_ for GSK2531401 [M13], which was comparable between participants with moderate hepatic impairment and healthy controls (GMR 1.0). In participants with mild hepatic impairment, C_max_ and AUC_0‐inf_ GMR were increased 1.3‐ to 2.0‐fold for all metabolites compared to matched healthy controls. The %CVb of systemic exposure for the predominant metabolites (C_max_, AUC_0‐t_, and AUC_0‐inf_) for participants with moderate hepatic impairment was somewhat higher (34.0%‒67.7%) than that observed for matched healthy controls (ranged between 23.0% and 32.3%) across all metabolites while the %CVb for participants with mild hepatic impairment was more similar (28.2%‒66.8%) compared with healthy matched controls (23.5%‒43.4%).

Results of the sensitivity covariate analysis reflected the primary statistical analysis, indicating that gender, age, and BMI had no significant impact on the assessment of hepatic impairment (data not shown).

Daprodustat was observed to be highly protein bound (>98%) to plasma proteins when determining the fraction unbound in the 3 hour postdose plasma samples in the participants with moderate hepatic impairment (mean 0.0034) and their matched healthy controls (mean 0.0028). In the participants with mild hepatic impairment and matched controls, the mean unbound fraction values were 0.0134 and 0.0032, respectively. Due to limited unbound daprodustat PK concentrations at 12 and 24 hours postdose, no formal statistical assessment of plasma protein binding was possible. There was a high degree of variability in the calculations of the unbound (free) PK parameters for daprodustat with %CVb values ranging from 156% to 578% for participants with hepatic impairment (Cohorts 1 and 3) and from 33.2% to 253% for matched controls (Cohorts 2 and 4). Unbound daprodustat exposure comparisons between the hepatic impairment groups and their matched controls reflected the comparisons of total daprodustat exposures, with GMR increases in peak and overall exposure (free C_max_, free AUC_0‐inf_) of 1.6‐ (90% CI 0.3‒8.0) and 2.2‐fold (90% CI 0.4‒11.7) observed in mild hepatic impairment and 2.3‐ (90% CI 1.1‒4.8) and 2.3‐fold (90% CI 1.1‒4.7) in participants with moderate hepatic impairment (Table 3). For the predominant metabolites, plasma protein binding was generally comparable across all cohorts within each metabolite and ranged from 58% to 78%. Unbound PK parameters were not calculated for the metabolites.

**Table 3:** Daprodustat Unbound Plasma Pharmacokinetic Parameters

	Part 1		Part 2	
Parameter	Moderate HI (n = 8)	Matched Controls (n = 8)	Mild HI (n = 7^a^)	Matched Controls (n = 6^a^, ^b^)
Free AUC_0‐inf_ (h•ng/mL)				
Arithmetic mean (SD)	1.6 (1.5)	0.4 (0.1)	11.8 (18.4)	3.6 (7.2)
Geometric mean (%CVb)	1.0 (160)	0.4 (33.2)	2.7 (578)	1.2 (225)
Ratio of geometric LS mean (90% CI)	2.3 (1.1, 4.7)		2.2 (0.4, 11.7)	
Free C_max_ (ng/mL)				
Arithmetic mean (SD)	0.7 (0.7)	0.2 (0.1)	3.8 (6.0)	2.1 (4.3)
Geometric mean (%CVb)	0.5 (156.3)	0.2 (54.4)	1.0 (475.2)	0.6 (253.1)
Ratio of geometric LS mean (90% CI)	2.3 (1.1, 4.8)		1.6 (0.3, 8.0)	

%CVb, between subject coefficient of variation; 90% CI, 90th percentile confidence interval; AUC_0‐inf_, area under the curve from zero to infinity; C_max_, maximum observed concentration; fu, unbound fraction; HI, hepatic impairment; LS, least squares; PK, pharmacokinetic; SD, standard deviation.

Unbound daprodustat PK parameters (free C_max_ and free AUC_0‐inf_) were derived by multiplying the PK parameter values for total daprodustat by the only calculable unbound fraction (fu) values obtained for each subject. All participants had only 1 calculable fu value (3 h sample) with the exception of one participant from Cohort 4 where the average of the 2 calculable fu values (3 and 12 h samples) was used since all but 1 participant had unquantifiable unbound concentrations at 12 and 24 h samples.

^a^ No free PK parameters could be estimated for 2 participants (mild HI and matched controls) due to all unbound concentrations for daprodustat being unquantifiable or nonreportable.

^b^ One participant from the matched controls for mild HI group was excluded from the table summaries due to missing PK endpoints.

### Pharmacodynamics

**Figure 2:**
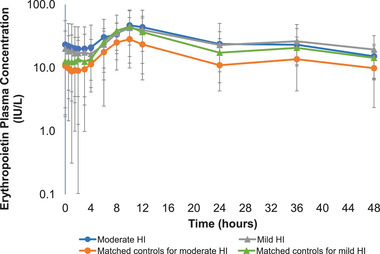
EPO plasma pharmacodynamic concentrations over time. EPO plasma concentrations following administration of a single 6‐mg dose of daprodustat on a semilogarithmic scale (mean ± standard deviation). The lower limit of quantification corresponds to 2.5 IU/L. HI, hepatic impairment.

Mean plasma concentration‐time profiles for EPO are displayed in Figure 2. The maximum EPO concentrations were achieved at median t_max_ values of 10 hours postdose across all cohorts (Table 4). EPO concentrations returned to baseline levels by approximately 48 hours postdose. In participants with hepatic impairment, systemic EPO exposure (AUC_0‐t, EPO_) was 1.2 (90% CI 0.8‒1.8) and 1.6‐fold (90% CI 1.0‒2.8) higher in the mild and moderate hepatic impairment groups than in the respective matched healthy controls; C_max_ EPO GMR increases between participants with mild and moderate hepatic impairment and the matched healthy controls were 1.1‐ (90% CI 0.7‒1.6) and 1.6‐fold (90% CI 1.0‒2.7) higher, respectively. When adjusting for baseline EPO values from each group, the baseline‐corrected AUC_0‐t, EPO_ from the mild and moderate hepatic impairment group GMR comparisons were between 0.3‐fold (GMR 0.7, 90% CI 0.3‒1.6) lower and 2.2‐fold (90% CI 1.0‒4.7) higher than the respective matched healthy volunteers, while the baseline corrected C_max_, EPO GMR comparisons were 0.2‐fold (GMR 0.8, 90% CI 0.5–1.5) lower and 1.5‐fold (90% CI 0.9‒2.6) higher for mild and moderate hepatic impairment groups, respectively (Table 4).

**Table 4:** EPO Plasma Pharmacodynamic Parameters

	Part 1		Part 2	
Parameter	Moderate HI (n = 8)	Matched Controls (n = 8)	Mild HI (n = 12)	Matched Controls (n = 9)
AUC_0‐t_, _EPO_ (h•IU/L)				
Arithmetic mean (SD)	1262 (1148)	698 (393)	1259 (683)	1062 (648)
Geometric mean (%CVb)	1002 (73.7)	611 (60.1)	1094 (60.7)	929 (57.1)
Ratio of geometric LS mean (90% CI)	1.6 (1.0–2.8)		1.2 (0.8–1.8)	
Baseline‐corrected AUC_0‐t_, _EPO_ (h•IU/L)				
Arithmetic mean (SD)	144 (450)	182 (145)	298 (557)	451 (246)
Geometric mean (%CVb)	266 (49.5)	123 (145)	270 (194)	387 (68.9)
Ratio of geometric LS mean (90% CI)	2.2 (1.0–4.7)		0.7 (0.3–1.6)	
t_max_, _EPO_ (h)				
Median (range)	10.0 (8.0–12.0)	10.0 (8.0–10.0)	10.0 (0–48)	10.0 (6.0–12.0)
C_max_, _EPO_ (IU/L)				
Arithmetic mean (SD)	48.9 (36.9)	28.4 (13.2)	43.9 (20.3)	45.9 (35.2)
Geometric mean (%CVb)	39.7 (75.9)	25.2 (60.7)	39.5 (52.7)	37.6 (71.1)
Ratio of geometric LS mean (90% CI)	1.6 (1.0–2.7)		1.1 (0.7–1.6)	
Baseline‐corrected C_max_, _EPO_ (IU/L)				
Arithmetic mean (SD)	25.6 (13.3)	17.7 (9.6)	23.9 (17.0)	33.2 (27.2)
Geometric mean (%CVb)	22.6 (59.9)	15.0 (74.3)	20.8 (89.7)	25.5 (86.7)
Ratio of geometric LS mean (90% CI)	1.5 (0.9–2.6)		0.8 (0.5–1.5)	

%CVb, between subject coefficient of variation; 90% CI, 90th percentile confidence interval; AUC_0‐t_, area under the curve from time zero (predose) to last time of quantifiable concentration; C_max_, maximum observed concentration; EPO, erythropoietin; HI, hepatic impairment; LS, least squares; SD, standard deviation; t_max_, time of occurrence of C_max_.

### Safety and Tolerability

Reported AEs were low in this study, and no AEs were determined to be study drug‐related. In Part 1, 3 of 16 participants (19%) experienced 1 AE each (one participant [13%] in Cohort 1 [moderate hepatic impairment] with skin abrasion and two participants [25%] in Cohort 2 [matched healthy control], 1 with upper respiratory tract infection and 1 with toothache). No AEs were reported in Part 2 of the study (mild hepatic impairment and matched controls). There were no deaths, serious AEs, AEs leading to withdrawal, or apparent differences in safety between the groups. There were no clinically relevant findings with regard to laboratory evaluations, vital signs, or ECGs.

## Discussion

The purpose of this study was to assess the effects of moderate and mild hepatic impairment on the PK, PD, and tolerability of daprodustat following a single 6‐mg dose in order to better guide administration of daprodustat in CKD patients who also have hepatic impairment. Daprodustat was rapidly absorbed in all groups (t_max_ 1.5‒2.0 hours) and the elimination t_1/2_ was similar among the hepatic impairment participants and controls (4.2‒4.7 hours). Overall exposure (AUC_0‐t_) to daprodustat was increased in participants with moderate and mild hepatic impairment compared to matched healthy controls. Peak exposure (C_max_) was increased in participants with moderate hepatic impairment and comparable in mild hepatic impairment compared with matched healthy controls. Unbound differences in daprodustat exposure reflected total daprodustat exposure, consistent with the similar values of albumin concentrations across the four cohorts (Table 1). It should be noted that the protein binding unbound fraction values were extremely variable and no statistical comparisons could be made.

Exposure results for all metabolites generally reflected those of the parent drug, whereby exposure was somewhat greater in the hepatic‐impaired subjects versus their matched controls (1.3–2.0‐fold greater). Increases were observed in both hepatic impaired groups, indicating that the enzymes responsible for metabolizing daprodustat were not meaningfully affected. This is consistent with reports of CYP enzymes having an extent of altered protein and activity levels in proportion to the severity of hepatic impairment.^23^ Additionally, in studies of liver samples from patients with cirrhotic livers, CYP2C8 proteins were unaltered versus control liver samples where other CYP enzymes are more susceptible to liver disease.^24^ Therefore, the modest increases in exposure of both daprodustat and its metabolites may be due to other pathophysiological effects of liver dysfunction such as reduced biliary excretion, reduced blood flow to the liver, the presence of intra‐ and extrahepatic portal‐systemic shunting, a capillarization of the sinusoids, or a reduction in the number and activity of the hepatocytes. These effects progress with the worsening of hepatic function.^25^

The baseline‐corrected EPO AUC_0‐t_ exposures were between 0.3‐fold lower and 2.2‐fold higher in the mild and moderate hepatic impairment groups, respectively, versus their matched controls. The baseline‐corrected EPO C_max_ exposures were similar between the hepatic impairment groups (25.6 and 23.9 IU/L) while their matched controls had a larger difference (17.7 vs 33.2 IU/L). It has been observed that EPO plasma concentrations are increased with the severity stage of hepatic impairment, as are markers of liver synthesis function such as albumin. Additionally, an inverse correlation with hemoglobin (anemia) was previously identified.^26^ There was evidence of higher baseline EPO concentrations in hepatic impairment subjects versus the healthy subjects in this study that were similar to values observed in Tacke et al. However, hemoglobin values and creatinine across the cohorts were in normal ranges, suggesting similar renal function in each hepatic impaired group and their matched controls (Table 1). As there was high variability of baseline corrected EPO exposures (%CVb range ∼50%–194%) within cohorts and considerable overlap of EPO versus time profiles among the cohorts (Figure 2), increased daprodustat exposures in the hepatic impairment cohorts did not appear to have a considerable impact on EPO levels. Although it is difficult to make definitive projections of how the single‐dose EPO findings from this study would translate to CKD patients with anemia and hepatic impairment on long‐term daprodustat treatment, routine monitoring of hemoglobin is expected, and doses titrated for each individual.

It was observed that the derived PK parameters differed between the two healthy control cohorts and that daprodustat exposure in healthy controls matched to moderate impairment (Cohort 2) was lower than what has been observed in other studies with healthy volunteers.^17^ The reason for lower exposure in this healthy cohort is unknown. If daprodustat exposures were similar to historical data (or healthy controls matched to mild impairment [Cohort 4]), the true effect of moderate hepatic impairment may be less than the 2.0‐fold exposure observed in this study.

No new safety concerns were identified in participants with mild or moderate hepatic impairment treated with a single oral 6‐mg dose of daprodustat.

A limitation of this study is that participants with severe hepatic impairment were not evaluated and daprodustat is primarily eliminated by the liver. Thus, the results of this study cannot be extrapolated to those with severe hepatic impairment. Additionally, lower‐than‐expected daprodustat exposure in healthy controls matched to moderate impairment (Cohort 2) may have compounded the interpretation of PK results.

In conclusion, peak and overall exposure to daprodustat were increased in participants with moderate and mild hepatic impairment compared to matched healthy controls; however, no meaningful differences in EPO were observed and no new safety concerns were identified.

## Conflicts of Interest

KMM, BCS, SA, ARC, and ACL are employees of and hold stock in GlaxoSmithKline (GSK). BR is a former employee of GSK. SC is a former employee of GSK and holds stock in GSK.

## Author Contribution

KMM, BR, SA, SC, ACL, and ARC contributed to the concept and design of the study. SA contributed to the acquisition of data. KMM, BCS, BR, SA, SC, ACL, and ARC contributed to the data analysis and interpretation. All authors provided critical comments throughout the draft development, provided final approval for submission and agree to be accountable for the work.

## Supporting information

Supporting Information
