# Supplementary material for: Pharmacokinetics of Daprodustat and Metabolites in Individuals with Normal and Impaired Hepatic Function
Source: Clin Pharmacol Drug Dev. 2022 Mar 30;11(5):562–75. doi: 10.1002/cpdd.1090 (PMC9310628; doi:10.1002/cpdd.1090)
Supplement: Supplementary file 1 — Supporting Information [file CPDD-11-562-s001.docx]

# Supplementary Information

**Supplementary Methods**

**Complete Exclusion Criteria**

Individuals were excluded from the study if any of the following criteria applied:

1. Electrocardiogram QT interval corrected for heart rate (QTc) using Fridericia’s formula (QTcF) >500 msec
2. Recent history of deep vein thrombosis, pulmonary embolism, or other thrombosis-related condition
3. Myocardial infarction or acute coronary syndrome or stroke or transient ischemic attack within the 12 weeks prior to enrollment
4. A pre-existing condition (other than a liver disease) interfering with normal gastrointestinal anatomy or motility that could have interfered with the absorption, metabolism, and/or excretion of daprodustat
5. Underwent cholecystectomy within the past 3 months
6. Chronic inflammatory joint disease (e.g., scleroderma, systemic lupus erythematosus, rheumatoid arthritis)
7. History of malignancy within the prior 2 years or known kidney mass >3cm (end-stage renal disease participants only) or currently receiving treatment for cancer (Note: The only exception was localized squamous cell or basal cell carcinoma of the skin definitively treated 12 weeks prior to enrollment)
8. Class IV heart failure, as defined by the New York Heart Association functional classification system
9. Current enrollment or past participation (i.e., administration of the last dose of investigational study drug) within the last 30 days (or 5 half-lives, whichever was longer) before Day 1 in this or any other clinical study involving an investigational study drug or any other type of medical research

Additional exclusion criteria for hepatically impaired individuals:

1. Presence of 8 × upper limit of normal elevations in aspartate aminotransferase, alanine aminotransferase, or bilirubin
2. Any other medical condition which, in the judgment of the investigator and medical monitor, could have jeopardized the integrity of the data derived from that subject or the safety of the subject
3. Advanced ascites (Grade 3)
4. Refractory encephalopathy as judged by the investigator or significant central nervous system disease (e.g., dementia or seizures) which the investigator considered would have interfered with the informed consent, conduct, completion, or results of this trial or constituted an unacceptable risk to the subject
5. Functional transjugular intrahepatic portosystemic shunt placement
6. Presence of hepatopulmonary or hepatorenal syndrome
7. Presence of primarily cholestatic liver diseases
8. History of liver transplantation
9. Signs of active infection, including active spontaneous bacterial peritonitis
10. Unstable cardiac function or hypertension with uncontrolled blood pressure (based on the investigator’s discretion)
11. Individuals with diabetes whose diabetes was not controlled (based on the investigator’s discretion)

Additional exclusion criteria for healthy controls:

1. Exposure to more than 4 new chemical entities within 12 months prior to the first dosing day
2. Presence of hepatitis B surface antigen at screening or positive hepatitis C antibody test result at screening or within 3 months before the first dose of study drug
3. Positive pre-study drug or alcohol screen
4. Positive human immunodeficiency virus antibody test
5. Regular use of known drugs of abuse
6. Regular alcohol consumption within 6 months prior to the study, defined as an average weekly intake of >14 drinks. One drink was equivalent to 12 g of alcohol: 12 ounces (360 mL) of beer, 5 ounces (150 mL) of wine, or 1.5 ounces (45 mL) of 80 proof distilled spirits. One unit was equivalent to 8 g of alcohol: a half-pint (~240 mL) of beer, 1 glass (125 mL) of wine, or 1 (25 mL) measure of spirits
